# Light‐Gated Thermal Domains in Nano‐Lanterns: Confined Heat Hotspots Sparks Electron Localization for Water Purification

**DOI:** 10.1002/advs.202513730

**Published:** 2025-10-30

**Authors:** Miao Fang, Zhiyuan Ning, He Guo, Xiaoteng Fan, Guodong Zhang, Qiuling Ma, Jian Zhou, Tiecheng Wang, Sihui Zhan

**Affiliations:** ^1^ State Key Laboratory of Soil and Water Conservation and Desertification Control College of Natural Resources and Environment Northwest A&F University Yangling Shaanxi Province 712100 P. R. China; ^2^ College of Biology and the Environment Nanjing Forestry University Nanjing 210037 P. R. China; ^3^ College of Animal Science and Technology Northwest A&F University Yangling 712100 P. R. China; ^4^ School of Environmental Science & Engineering Tianjin University Tianjin 300350 P. R. China

**Keywords:** heat‐confinement, nanoconfinement, non‐radical, peracetic acid, sulfadiazine

## Abstract

Peroxyacetic acid (PAA) oxidation technology receives widespread concerns for water purification with minimal secondary pollution. Conventional heat‐driven PAA activation generally wastes energy during solution heating. In this study, “light‐gated thermal domains” concept is developed in a hollow porous carbon nanosphere (HPCS), and the confined heat hotspots lead to a higher temperature (80 °C) in the internal space than in the solution (40 °C). This endows the HPCS with exceptional redox capacity, optical response, and electron transfer capability. The degradation efficiency of sulfadiazine in the HPCS+PAA catalytic system reached more than 98% within 90 min of irradiation, with a reaction rate constant 11 times higher than that in the non‐confined system. The “light‐gated thermal domains” induces electron localization and decreases PAA activation energy barriers. In contrast to the non‐confined system dominated by the radical oxidation pathway, heat‐confinement exhibits synergies between the radical and non‐radical pathways, enabling rapid pollutant degradation. Zebrafish embryo experiments validated the pollutant detoxification capabilities of this system. This “light‐gated thermal domains” ensures long‐lasting robustness of PAA activation and paves a novel way for the development of sustainable catalytic water purification technologies.

## Graphical abstract

A pore heat‐confined hollow porous canbon nanosphere catalyst is synthesized, with superior photo‐induced heat/redox response, revolutionizing peroxyacetic acid (PAA) activation for sustainable water purification. It overcomes traditional thermal PAA oxidation's energy inefficiency, enabling rapid pollutant degradation with a rate constant 11 times higher than nonconfined system, underscoring its remarkable superiority and potential for practical applications.

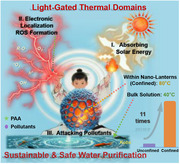

## 1 Introduction

Peroxyacetic acid (PAA, CH_3_COOOH), a potent peroxyacid oxidizer, is extensively utilized in the food and healthcare industries.^[^ ^1^ ^]^ The high efficiency of PAA in the inactivation of pathogenic bacteria and the low generation of toxic byproducts make it a promising disinfectant for wastewater.^[^ ^2^ ^]^ PAA oxidation was developed to eliminate contaminants such as phenols, dyes, and antibiotics from wastewater.^[^ ^3^ ^]^ Homolytic cleavage of the O−O bond in PAA can produce oxidizing species ·OH and organic radicals R‐O· (including CH_3_COO· and CH_3_COOO·).^[^ ^4^ ^]^ These oxidizing species have been proven to play a significant role in the elimination of stubborn organics.^[^ ^5^ ^]^ The bond energy of the peroxide bond (159 kJ mol^−1^) in the PAA molecule is much weaker than those of peroxymonosulfate (317 kJ mol^−1^) and H_2_O_2_ (213 kJ mol^−1^).^[^ ^2^ ^]^ The lower bond energy of PAA renders the thermodynamic formation of reactive radicals more feasible. The contributions of different reactive radicals to the degradation of organic pollutants largely depend on the PAA activation methods.^[^ ^6^ ^,^ ^7^ ^]^

Recent studies have focused on the activation of PAA using exogenous energy (e.g., solar energy) and/or catalysts to produce reactive radicals.^[^ ^2^ ^]^ Thermal materials can absorb light energy and induce heat conversion, thereby increasing the reaction temperature.^[^ ^8^ ^]^ Increasing the reaction temperature improves the reaction rate. Higher reaction temperatures have been demonstrated to overcome reaction activation barriers and enhance product selectivity.^[^ ^9^, ^10^ ^]^ Thermocatalytic oxidation reactions can occur when the temperature exceeds the light‐off temperature for thermocatalytic oxidation.^[^ ^11^ ^]^ Therefore, heat conversion is an alternative method for PAA activation. Some heating materials (semiconductor materials, carbon, precious metals, etc.) can absorb and convert irradiation into heat energy.^[^ ^12^ ^]^ This generated heat is then driven by a temperature gradient to diffuse into the solution. However, most of the heat energy is generally consumed in heating the solution rather than generating localized high temperatures.^[^ ^13^ ^]^ Consequently, it is important to develop a PAA activation system that can efficiently utilize the heat generated by heat conversion.

Recently, nanoconfinement reactors have been developed for adsorption, catalysis, separation, energy storage, and biomedical fields.^[^ ^14^, ^15^ ^]^ The confinement effect can provide a critical diffusion length scale for the encapsulation of oxidizing species and reactants.^[^ ^16^ ^]^ Nanoconfinement materials can enhance the reaction rate and selectivity of pollutant degradation.^[^ ^17^ ^]^ Current studies on nanoconfinement catalysis for environmental remediation include nanoconfinement photocatalysis, nanoconfinement electrocatalysis, and nanoconfinement membrane catalysis, which significantly improve the reaction kinetics for pollutant removal.^[^ ^15^ ^]^ Hollow porous carbon nanospheres (HPCS) are a new type of heat‐active nanomaterials, and their internal spaces serve as localized sites for reaction heat and facilitate the dynamic movement of reactants and products, thereby enhancing reaction rates. The unique structure of HPCS also acts as a barrier that prevents interfering substances, such as natural organic matter from entering the internal reaction space.^[^ ^18^ ^]^ Therefore, a “light‐gated thermal domains” effect, a locally heat‐confined reaction space within HPCS under irradiation, was proposed to overcome reaction barriers of PAA activation. Nevertheless, the underlying mechanisms of “light‐gated thermal domains” within HPCS on organic pollutant degradation during PAA activation are still unclear.

In this study, the confined heat hotspots‐driven PAA activation by “light‐gated thermal domains” within HPCS rapidly achieved water purification, with a focus on the degradation of antibiotic sulfadiazine (SDZ). First, the HPCS was prepared, its optical‐electrochemical characteristics were monitored, and the temperature distribution and velocity vector for the single‐particle HPCS was visualized by ANSYS Fluent simulation to elucidate the locally heat‐confined reaction space. Then, the SDZ degradation performance of the HPCS+PAA system under different operational conditions was tested. Subsequently, the strengthening mechanisms induced by heat‐confinement effects, involving reactive radical formation and contributions, localization of electron densities, Gibbs free energies of PAA activation, and the SDZ decomposition process, were revealed. Finally, the sustainability and environmental impacts of the HPCS+PAA system were evaluated via zebrafish embryo toxicity tests, continuous‐flow experiments, and pollutant removal. This work revolutionizes PAA activation for sustainable water purification and overcomes the energy inefficiency of traditional thermal PAA oxidation processes, aiming to expand this technology from laboratory‐scale to full‐scale application in water purification.

## 2 Results and Discussion

**Figure 1:**
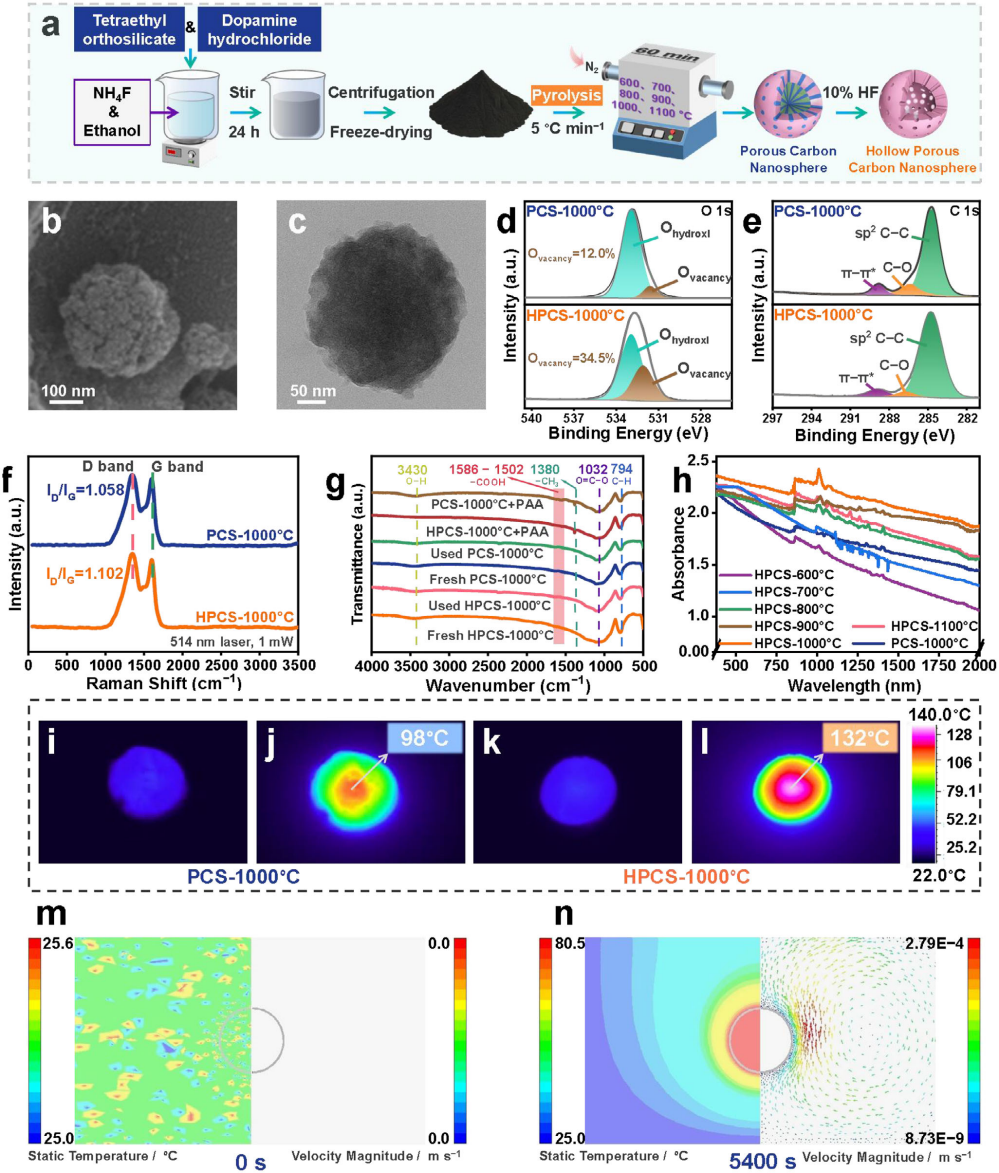
Characterization of the catalysts. a) Preparation process of materials. b) SEM image of HPCS‐1000 °C. c) TEM image of HPCS‐1000 °C. d) O1s and e) C1s of XPS. f) Raman spectra of catalysts. g) FTIR spectra of catalysts before and after reaction. h) Irradiation absorbance of catalysts. Thermal imagery of PCS‐1000 °C under i) dark and j) irradiation for 60 min; thermal imagery of HPCS‐1000 °C under k) dark and l) irradiation for 60 min. Numerical simulation of temperature distribution and velocity vector for HPCS at the reaction time m) 0 s and n) 5400 s.

The materials were prepared as shown in **Figure** **1**a. The structure and morphology of HPCS‐1000 °C and PCS‐1000 °C were characterized. The PCS‐1000 °C and HPCS‐1000 °C exhibited spherical morphology with average diameters of ≈300 nm (Figures 1b,c; S1a,b, Supporting Information). High‐resolution Transmission Electron Microscope (HRTEM) images of the HPCS‐1000 °C and PCS‐1000 °C also exhibited spherical morphology with abundant mesoporous (Figure S1c,d, Supporting Information). The amorphous structures of PCS‐1000 °C and HPCS‐1000 °C were demonstrated by the selected area electron diffraction patterns (the inset of Figure S1c,d, Supporting Information). C, O, N, and Si elements were distributed uniformly in the HPCS‐1000 °C and PCS‐1000 °C, and an obvious decrease in the intensity of Si element in the HPCS‐1000 °C was observed compared with that in the PCS‐1000 °C (Figure S1e−h, Supporting Information). After the removal of the Si element, the specific surface area of the HPCS‐1000 °C was 683.3 m^2^ g^−1^, which was much higher than 125.0 m^2^ g^−1^ of the PCS‐1000 °C (Table S1, Supporting Information). A higher specific surface area exposed more active sites, thereby favoring PAA activation. The N_2_ adsorption‐desorption isotherms of the HPCS‐1000 °C and PCS‐1000 °C presented a combination of type I and type IV isotherms (Figure S2, Supporting Information),^[^ ^19^, ^20^ ^]^ revealing the presence of microporous and mesoporous structures.^[^ ^21^ ^]^ More mesoporous structures were observed about *c.a*. 3.4 nm in the HPCS‐1000 °C compared to *c.a*. 2.5 nm of the PCS‐1000 °C, favorable for the entry and exit of reactants and products into the interior of the materials during the chemical reactions.^[^ ^22^ ^]^

As analyzed by X‐ray photoelectron spectroscopy (XPS) (Figure 1d), two peaks located at 532.5 and 533.7 eV were deconvolved corresponding to oxygen vacancies (O_V_) and surface‐adsorbed oxygen species, respectively, and the peak of O_V_ in HPCS‐1000 °C was nearly 3 times higher (34.5%) than that in PCS‐1000 °C (12.0%) (Figure S3, Supporting Information). As the calcination temperature increased, the O_V_ density in the HPCS increased (Figure S4, Supporting Information).^[^ ^23^, ^24^ ^]^ O_V_ can transfer electrons to PAA and activate PAA to generate a variety of radicals, and increased O_V_ facilitates the adsorption and degradation of pollutants on the surface of the catalysts.^[^ ^9^ ^]^ Three carbon species assigned to C−O (286.9 eV), sp^2^ C−C (284.5 eV), and π−π* (289.1 eV) were deconvolved (Figure 1e) and a relatively higher abundance of π−π* in the HPCS‐1000 °C demonstrated that HF etching enhanced the graphitization of the materials.^[^ ^14^ ^]^ As shown in Figure 1f, two peaks at 1590 cm^−1^ (G band) and 1360 (D band) in the Raman spectrum were observed, and the HPCS‐1000 °C exhibited more deficiency sites than PCS‐1000 °C because of its higher I_D_/I_G_ ratio.^[^ ^25^ ^]^ As analyzed by Fourier transform infrared spectroscopy (FTIR) (Figure 1g), the fresh and used HPCS‐1000 °C and PCS‐1000 °C materials had similar peaks. In addition, the HPCS‐1000 °C and PCS‐1000 °C materials had strong optical absorption abilities in the spectral range of 400‐2000 nm (Figure 1h). This strong light absorption capacity contributed to a strong heat effect. In Figure 1i,j, the surface temperature of the PCS‐1000 °C reached 98 °C after 60 min of exposure to the Xenon lamp (AM1.5G, 500 mW cm^−2^), while that of the HPCS‐1000 °C was up to 132 °C (Figure 1k,l). Correspondingly, an obvious increase in the bulk solution temperature was detected when the HPCS was illuminated, further illustrating the occurrence of the heating process (Figure S5, Supporting Information). Because the zeta potentials of HPCS and PCS were not significantly different (Figure S6, Supporting Information), the absorptive capacity of HPCS was probably attributed to the internal voids. Figure 1m,n depicts the temperature changes and velocity vectors in the internal voids of a single HPCS particle. The water temperature around the HPCS was homogenous and equivalent to the initial environmental temperature (25 °C, 0 s), as shown in Figure 1m. After 90 min irradiation, the temperature inside the HPCS void space reached 80 °C (Figure 1n), while the temperature of the bulk solution decreased gradually with increasing distance from the HPCS surface. These simulation results demonstrated that the HPCS exhibited superior optical‐induced heat properties.

Figure S7 (Supporting Information) depicts the SDZ degradation performance of the PAA oxidation systems activated by the PCS and HPCS catalysts prepared at different calcination temperatures. The SDZ degradation efficiency of the HPCS+PAA system was significantly higher than that of the PCS+PAA system at all selected calcination temperatures. These results could be attributed to the local high temperature generated inside the confined materials, which promoted PAA activation and reaction rates. The calcination temperature of HPCS significantly influenced the SDZ degradation by activating PAA. When the calcination temperature increased from 600 to 1000 °C, the SDZ degradation efficiency was enhanced from 23.9% to 48.7% within 60 min, while it decreased by 25.7% at 1100 °C (Figure S8, Supporting Information). These phenomena could be attributed to the decreased optical‐absorption ability of HPCS‐1100 °C (Figure 1h) owing to the agglomeration of catalysts.^[^ ^26^ ^]^ This trend in catalytic activity was consistent with the measured activation rate constant for PAA, which also increased at higher calcination temperatures (up to 1000 °C). This agreement strongly supported the conclusion that O_V_ density was a key factor governing PAA activation (Figure S9; Table S2, Supporting Information).^[^ ^27^, ^28^ ^]^ Consequently, HPCS‐1000 °C was chosen for the following experiments. Relatively lower SDZ initial concentrations favored its degradation, and ≈86.5% of SDZ was degraded within 90 min in the HPCS+PAA system under the SDZ initial concentration of 1 mg L^−1^ (Figure S10, Supporting Information). In addition, the increase in the catalyst dosages from 100 to 300 mg L^−1^ significantly promoted the SDZ degradation both in the HPCS+PAA and PCS+PAA systems, while no significant changes in the SDZ degradation efficiency after 90 min treatment were observed between the catalyst dosages of 300 and 400 mg L^−1^ (Figure S11, Supporting Information). The following studies were performed at the catalyst dosage of 300 mg L^−1^.

**Figure 2:**
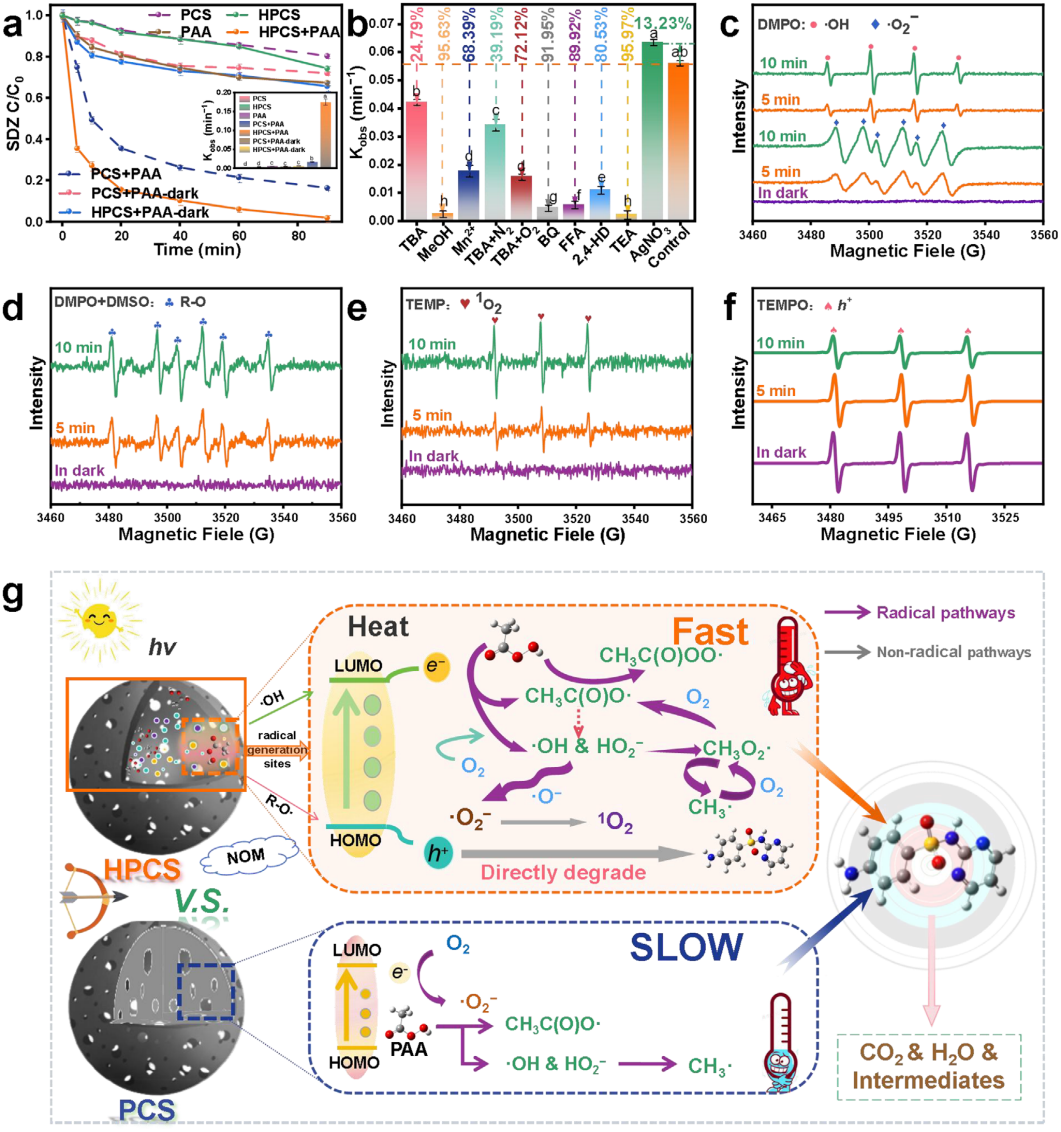
Catalytic performance and removal mechanism. a) SDZ degradation performance in various reaction systems. b) SDZ degradation kinetics under different ROS scavengers. (*p* < 0.05, Error bars: ± SD, *n* = 3). EPR profiles of c) ·OH and ·O2^−^, d) R‐O· radicals, e) ^1^O_2_, and f) photogenerated holes in the HPCS + PAA oxidation system. g) SDZ degradation mechanisms in different oxidation systems. Experimental conditions: [CSs] = 300 mg L^−1^, [PAA] = 227.3 µmol L^−1^, [SDZ] = 1 mg L^−1^, calcination temperature = 1000 °C, treatment time = 90 min, and pH 7.0.

The SDZ degradation performances of various reaction systems were compared in **Figure** **2**a. PAA could be directly activated under irradiation with a slow rate constant of merely 9.18 × 10^−4^ min^−1^ (Figure S12, Supporting Information), and only 32.6% of SDZ was degraded after 90 min. The removal efficiencies of SDZ were 25.7% for HPCS‐1000 °C and 19.6% for PCS‐1000 °C alone after 90 min, associating with the adsorption properties. HPCS‐1000 °C individually could also activate PAA in the dark, and then 34.4% of SDZ was degraded after 90 min. Under irradiation, PAA was rapidly decomposed in the HPCS+PAA system with the kinetic rate constant 2.1 times higher than that in the PCS+PAA system (Figure S12, Supporting Information). Correspondingly, the HPCS+PAA system achieved over 98% degradation of SDZ within 90 min, exhibiting a pseudo‐first‐order rate constant (0.1744 min^−1^) that was 11 times greater than that of the PCS+PAA system (0.0159 min^−1^). These results further prove that heat‐driven nanoconfinement led to the activation of PAA and SDZ degradation. The higher graphitization favored more sp^2^ structures and π‐band in materials, which benefited the heat conversion.^[^ ^29^, ^30^ ^]^ Thus, the HPCS‐1000 °C with high graphitization structures enhanced heat conversion performance, which promoted the PAA activation and the SDZ degradation. Moreover, the results demonstrated that the pure thermal effect primarily accelerated the reaction, whereas the photochemical process produced a synergistic enhancement (Figures S13 and S14, Supporting Information). A brief comparison of organic pollutant degradation by catalytic PAA oxidation is listed in Table S3 (Supporting Information). The catalytic performance of HPCS+PAA under irradiation was satisfactory.

The mechanisms of enhanced SDZ degradation in the HPCS+PAA system were explored in terms of ROS formation, electron transfer, electron localization, and PAA activation energy. Previous studies reported that various carbon‐containing radicals including CH_3_CO_3_·, CH_3_CO_2_·, CH_3_O_2_·, and CH_3_· could be generated during PAA activation (Equations (1), (2), (3), (4), (5), (6), (7), (8)),^[^ ^31^, ^32^, ^33^, ^34^, ^35^ ^]^ and ·OH, ·O_2_^−^, and ^1^O_2_ would also be formed in this case:

(1) $$\pi -\mathrm{electrons}+CH_{3}\mathrm{COOOH}\to CH_{3}CO_{2}\cdot +OH^{-}$$

(2) $$CH_{2}O^{-}+CH_{3}\mathrm{COOOH}\to CH_{2}O+CH_{3}CO_{2}\cdot +OH^{-}$$

(3) $$\begin{array}{ccc} & & \mathrm{Carbon}\;\mathrm{interior}-OH^{-}+CH_{2}\mathrm{COOOH}\to CH_{3}CO_{2}\cdot \\ & & \;+\;\mathrm{Carbon}\;\mathrm{interior}-O\cdot^{-}+H_{2}O \end{array}$$

(4) $$\begin{array}{ccc} & & \mathrm{Carbon}\;\mathrm{interior}-\mathrm{OO}H^{-}+CH_{3}\mathrm{COOOH}\to CH_{3}CO_{2}\cdot \\ & & \;+\;{\mathrm{Carbon}\;\mathrm{interior}-O}_{2}^{-}\cdot +H_{2}O \end{array}$$

(5) $$CH_{3}CO_{2}\cdot +CH_{3}\mathrm{COOOH}\to CH_{3}CO_{3}\cdot +CH_{3}\mathrm{COOH}$$

(6) $$CH_{3}CO_{2}\cdot \to CH_{3}\cdot +CO_{2}$$

(7) $$CH_{3}\cdot +O_{2}\to CH_{3}O_{2}$$

(8) $${\mathrm{Carbon}\mathrm{interior}-O}_{2}^{-}+\pi -\mathrm{electrons}\to \mathrm{Carbon}\mathrm{interior}{-}^{1}O_{2}$$

Figures 2b and S15 (Supporting Information) depict the SDZ degradation performances in the HPCS+PAA system with the addition of different radical scavengers. The presence of tert‐butanol (TBA) as a ·OH scavenger inhibited the SDZ degradation and the reaction rate constant decreased by 24.79% (Figure S15a, Supporting Information; Figure 2b). Methanol (MeOH), as a scavenger for both ·OH, CH_3_CO_3_·, and CH_3_CO_2_,^[^ ^31^ ^]^ sharply inhibited SDZ degradation and the reaction rate constant decreased by 95.63%, indicating that CH_3_CO_3_· and CH_3_CO_2_· probably played critical roles in the SDZ degradation process (Figure S15b, Supporting Information; Figure 2b). Mn^2+^ and 2,4‐hexadienoic acid (2,4‐HD) were used as quenching agents for CH_3_CO_3_· and CH_3_CO_2_·,^[^ ^32^ ^]^ and the SDZ degradation rate constant decreased by 68.39% and 80.53%, respectively (Figure S15c,d, Supporting Information; Figure 2b), further confirming the significant roles of CH_3_CO_3_· and CH_3_CO_2_·. The SDZ degradation rate constant decreased by 72.12% and 39.19% under oxygen‐enriched and anaerobic conditions in the presence of TBA (Figure S15e, Supporting Information; Figure 2b), suggesting that CH_3_O_2_· also had a significant role in SDZ degradation.^[^ ^33^ ^]^ Concomitantly, the degradation performance of SDZ increased significantly under oxygen‐enriched conditions and decreased under anaerobic conditions when oxygen or nitrogen was introduced (Figure S16, Supporting Information). This result evidenced that oxygen participated in the generation of CH_3_O_2_·, thus enhancing SDZ degradation.^[^ ^31^ ^]^ Zhou et al. also proved the important roles of CH_3_CO_3_·, CH_3_CO_2_·, and CH_3_O_2_· radicals in the degradation of organic pollutants.^[^ ^34^ ^]^ As shown in Figures 2b and S15f (Supporting Information), the presence of benzoquinone (BQ) led to a 91.95% decline in the SDZ degradation rate constant, proving the significant contribution of ·O_2_.^−[^ ^35^ ^]^ When 5,5‐dimethyl‐1‐pyrrolidinium‐N‐oxide (DMPO) was added to the HPCS+PAA oxidation system, the signals of DMPO‐·OH and DMPO‐·O_2_^−^ were also detected (Figure 2c). In addition, the signals of carbon‐containing radicals were observed after the addition of DMPO and DMSO, and the signal intensity was intensified as the reaction time continued (Figure 2d), as analyzed by electron spin resonance spectrometer (EPR). Thus, it was evident that CH_3_CO_3_·, CH_3_CO_2_·, CH_3_O_2_·, ·OH, and ·O_2_^−^ all played significant roles in the degradation of SDZ (Table S4, Supporting Information).

Furfuryl alcohol (FFA) as a quenching agent for ^1^O_2_ was added to the HPCS+PAA system and the SDZ degradation rate constant decreased by 89.92% (Figures 2b; S15g, Supporting Information), suggesting its important role.^[^ ^36^ ^]^ The electrons and holes photogenerated in the photocatalytic process migrated to the interior of the catalyst and participated in redox reactions,^[^ ^10^ ^]^ whereas the recombination of electrons and holes greatly reduced the rate of redox reactions (Equations 9 and 10). Therefore, the addition of AgNO_3_ increased the SDZ degradation rate by 13.23%, as shown in Figures 2b and S15h (Supporting Information). AgNO_3_ can capture electrons and extend the lifetime of *h*.^+[^ ^37^, ^38^ ^]^ When triethanolamine (TEA) was added to the reaction system to capture *h* ^+^, the SDZ degradation rate constant dramatically decreased by 95.97% (Figures 2b; S15i, Supporting Information), further proving the decisive role of *h* ^+^ oxidation. The important role of *h* ^+^ oxidation in pollutant degradation was demonstrated by Carr et al.^[^ ^38^ ^]^ Typical 2,2,6,6‐tetramethylpyridine (TEMP)‐^1^O_2_ signals with an intensity ratio of 1:1:1 (Figure 2e) were detected when TEMP was added as a trapping agent for ^1^O_2_.^[^ ^36^ ^]^ TEMPO signals were also observed in the dark, and their signals gradually decreased with increasing reaction time under irradiation (Figure 2f), confirming the reactions between *h* ^+^ and TEMPO. Thus, *h* ^+^ and ^1^O_2_ were assumed to be the primary non‐radical pathways for SDZ degradation in the HPCS+PAA system (Table S4, Supporting Information), which proceeded via a synergistic mechanism together with the radical pathways.

(9) $$\mathrm{Carbon}+hv\;\to e^{-}+h^{+}$$

(10) $$e^{-}+h^{+}\;\to \;hv+\mathrm{electron}-\mathrm{hole}\;\mathrm{pairs}$$

As a control, the SDZ degradation performances in the PCS+PAA system with the addition of different scavengers were also evaluated, as shown Figure S17 (Supporting Information). The contributions of the non‐radical pathways for SDZ degradation were quite weak, and ·OH, CH_3_CO_2_·, and ·O_2_^−^ dominated the SDZ degradation processes via radical oxidation pathways (Figure S17c, Supporting Information).

The degradation mechanisms of SDZ in the different oxidation systems under irradiation are illustrated in Figure 2g. In the HPCS+PAA system, the heat‐confined materials enhanced solar energy absorption, which generated localized heat energy inside and around the catalysts and accelerated chemical reactions. Meanwhile, the HPCS catalyst exhibited a high activation efficiency for PAA; different types of radicals and non‐radicals were generated in situ, and satisfactory SDZ degradation performance was realized through the dual effects of radical and non‐radical pathways. In contrast, in the PCS+PAA system, the radical pathway played a decisive role.

**Figure 3:**
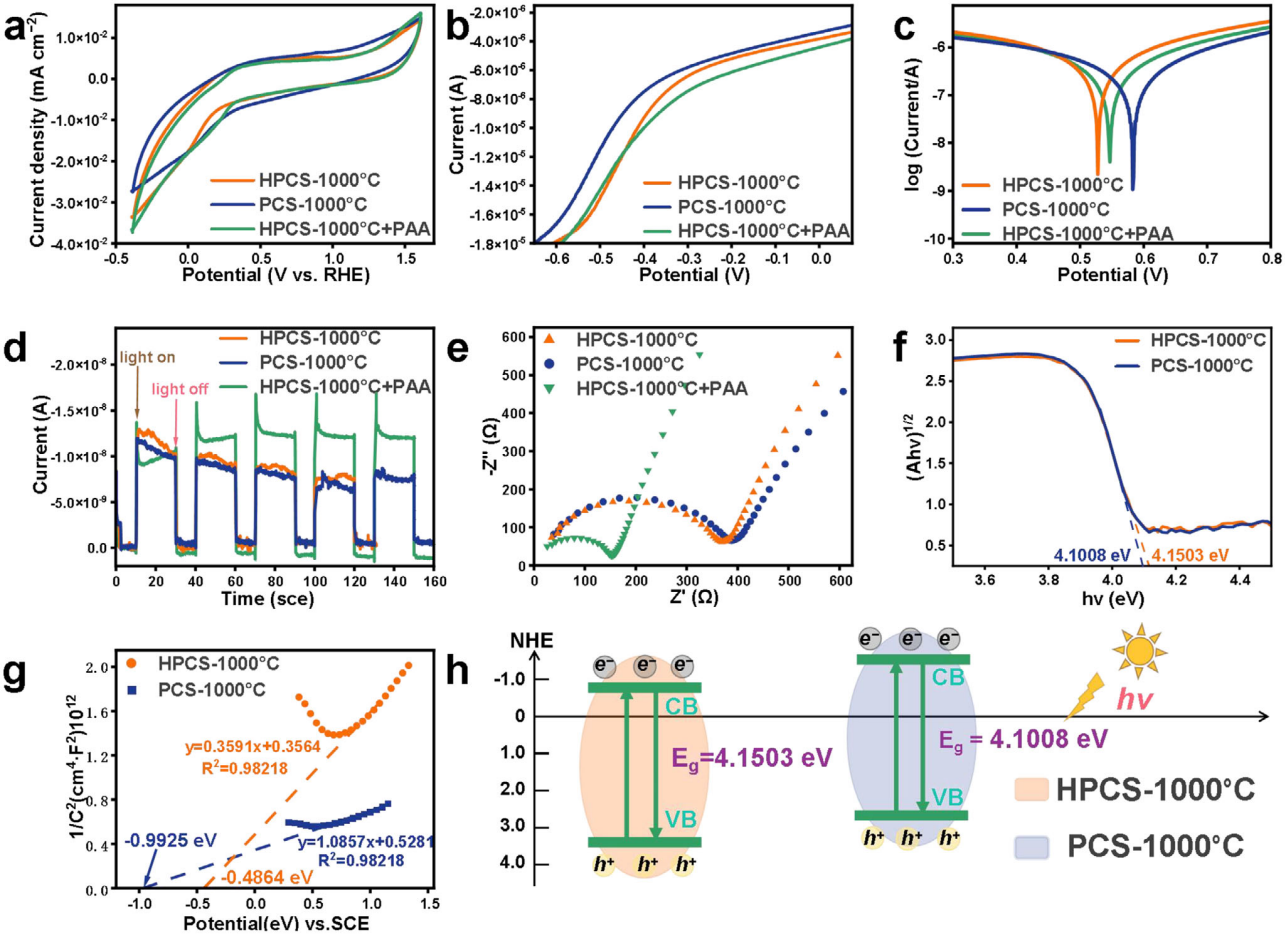
Photoelectrochemical characterization of catalysts. a) CV curves of catalysts. b) LSV curves. c) Tafel profiles. d) i‐t curves. e) Nyquist plots. f) Bandgaps. g) The Mott‐Schottky plots. h) Schematic diagram of CB and VB energy levels of HPCS‐1000 °C and PCS‐1000 °C.

Heat‐confinement favors electron transfer between nanoparticles.^[^ ^39^ ^]^ The electrochemical properties of HPCS‐1000 °C and PCS‐1000 °C materials were evaluated. The HPCS+PAA system exhibited a more favorable current response in the cyclic voltammetry (CV) curves (**Figure** **3**a), indicating a stronger electron transfer capability. The oxygen evolution potential (OEP) reflects the ability of the materials to perform oxidation reactions.^[^ ^40^ ^]^ The OEP (compared with RHE) of HPCS‐1000 °C+PAA under irradiation was higher than those of HPCS‐1000 °C and PCS‐1000 °C (Figure 3b). Therefore, the HPCS‐1000 °C+PAA showed the greatest activity in redox reactions. A lower free‐corrosion potential typically corresponds to a higher corrosion rate.^[^ ^39^ ^]^ The HPCS‐1000 °C+PAA exhibited narrower margins under irradiation compared to the HPCS‐1000 °C and PCS‐1000 °C, suggesting a faster electron transfer rate (Figure 3c). The photocurrent intensity of HPCS‐1000 °C+PAA was significantly higher than those of the HPCS‐1000 °C and PCS‐1000 °C (Figure 3d), indicating significantly improved charge separation in the HPCS‐1000 °C after the addition of PAA.^[^ ^41^ ^]^ The greatest electron transfer capacity for the HPCS‐1000 °C+PAA was also confirmed by the smallest radii in the electrochemical impedance spectroscopy in Figure 3e. Tauc plots were calculated from the solid‐state UV/vis‐IR absorption spectra, and the bandgaps of HPCS‐1000 °C and PCS‐1000 °C were 4.1503 and 4.1008 eV, respectively (Figure 3f). Based on the Mott‐Schottky plot (pH = 1), the conduction band (CB) potentials of HPCS‐1000 °C and PCS‐1000 °C were −0.4864 and −0.9925 eV versus SCE, respectively (Figure 3g). The calculated energy levels of the two catalysts are shown in Figure 3h. The valence band (VB) potential of HPCS‐1000 °C was more positive than that of PCS‐1000 °C, indicating a higher oxidation capability of the HPCS‐1000 °C.^[^ ^42^ ^]^ Generally, the HPCS‐1000 °C exhibited an excellent redox capacity, optical response performance, and strong electron transfer capability, which was expected to be a superior catalyst in the activation of PAA for the degradation of organic pollutants.

**Figure 4:**
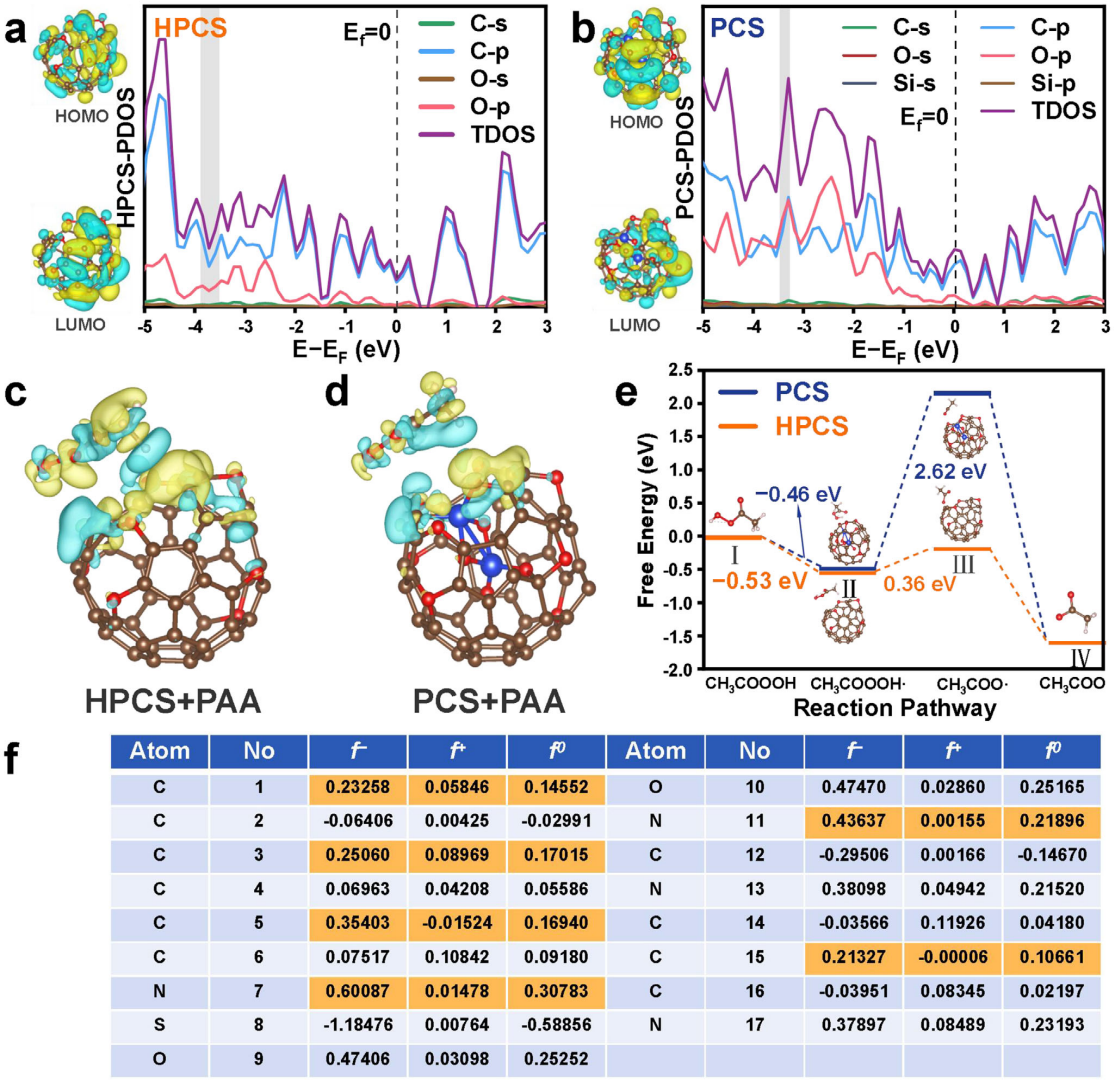
DFT calculations of nanospace constraint effects. PDOS of a) HPCS and b) PCS. Charge density difference mappings of c) HPCS and d) PCS. Blue color: charge depletion; yellow color: charge accumulation. e) Free energy of PAA activation by HPCS and PCS. f) Fukui function of SDZ.

Density functional theory (DFT) calculations were performed to analyze the microchemical states and electronic distributions, and the HPCS and PCS models were constructed based on the approximate elemental ratios (Figure S18, Supporting Information). First, the highest occupied molecular orbita (HOMO) and lowest unoccupied molecular orbital (LUMO) of HPCS and PCS were analyzed. According to the total density of states (TDOS) and partial wave density of states (**Figure** **4**a,b), the electron density of the HOMO was clustered in a narrow region, indicating a clear localization of the electron densities, and the electron density of the off‐domain occurred below the Fermi energy level (yellow region).^[^ ^43^ ^]^ The reduced energies of the HOMO and LUMO of HPCS compared to those of PCS indicated that HPCS could promote the delocalization of the electron density, which in turn enhanced the bonding with PAA molecules. The higher density of states in the 2p orbitals of the C and O atoms played a major role in PAA activation. Meanwhile, the Si (2s, 2p) of PCS behaved inertly, whereas the C_2p_ orbitals of HPCS played a major role, followed by O_2p_. HPCS exhibited a negatively shifted C_2p_ orbital with significantly increased density, suggesting enhanced electron delocalization that facilitated electron donation to the antibonding orbital of PAA. This charge transfer resulted in a weakened O–O bond, consistent with the reduced activation barrier observed in Figure 4e. The sp^2^‐hybridized C_2p_ orbitals contributed substantially to this effect via π‐backdonation into the σ^∗^orbital of PAA.^[^ ^44^ ^]^ Thus, the capture of reaction intermediates by the HPCS was enhanced and the degradation of pollutants was promoted.

The differences in the charge densities of HPCS and PCS adsorbed PAA are shown in Figure 4c,d. The HPCS‐adsorbed PAA had a higher charge density and greater electron gain and loss. This suggested a significant interaction between PAA and HPCS via chemisorption, further indicating that the heat‐confinement HPCS with a core‐shell structure regulated the electronic structure to form an active center.^[^ ^44^ ^]^ Figure 4e depicts the Gibbs free energies of PAA activation by the catalysts. More energy was released to form CH_3_COOOH· in the HPCS+PAA system (−0.53 eV) compared to the PCS+PAA system (−0.46 eV), indicating that the adsorption process of PAA on HPCS was more exothermic and spontaneous. Zhang et al. reported that reductive cleavage of the peroxide bond produced ·OH and −OH groups.^[^ ^45^ ^]^ The adsorbed PAA was activated for proton transfer and formed ·OH and CH_3_COO·, and ·OH was subsequently dissociated into H^+^ and ·O.^[^ ^46^ ^]^ The activation energy barriers experienced by HPCS and PCS from processes II to III were 0.36 and 2.62 eV, respectively. As a result, the dual reaction sites of adsorption and activation considerably shortened the migration distance of ·OH, which greatly improved the catalytic activity. These results also proved that the heat‐confined structure enhanced the electron density of HPCS and induced efficient activation of PAA.^[^ ^47^ ^]^

Charge distributions, including the molecular orbitals, electrostatic potentials, and Fukui functions of SDZ, were calculated using DFT, as shown in Figures 4f and S19 (Supporting Information). HOMO denotes the ability to supply electrons, whereas LUMO denotes the ability to receive electrons.^[^ ^48^, ^49^ ^]^ The entire SDZ molecule was electron sensitive except for 18H, 21H, and 24−27H (Figure S19a, Supporting Information). The HOMO of SDZ was distributed in and around atoms 1−10 and 18−23, mainly because of the sulfuryl and amino groups. 1C, 3C, 5C, 15C, 7N, and 17N had high *f* ^−^ and *f* ^0^ values (Figure 4f), indicating that these atoms were sensitive to attack by electrophilic oxygen species. The degradation intermediates of SDZ were further analyzed in Figure S20 (Supporting Information) and listed in Table S5 (Supporting Information), and the SDZ decomposition processes in the HPCS+PAA system under irradiation were proposed in Figure S21 (Supporting Information). The SDZ decomposition processes involved S−N bond breaking, hydroxylation, and nitrification. On the one hand, under the roles of radical and non‐radical oxidation, the S−N bond in SDZ molecules was ruptured to produce 2‐aminopyrimidine (P1) and *p*‐aminobenzene sulfonic acid (P2).^[^ ^50^ ^]^ Subsequently, byproduct P3 was generated and further oxidized to byproducts P4 and P5. Alternatively, the C−N bond in the byproduct P3 may break and lead to the production of the intermediate P6, which was subsequently decomposed to P7 via a ring‐opening process. On the other hand, hydroxylation of SDZ could produce the byproduct P8, which was then decomposed to P1 and P2 after the cleavage of the S−N bond. Byproduct P9 was generated via the hydroxylation of SDZ, which was then further decomposed into intermediates P10, P11, and P12. These results agree with the the HOMO and Fukui index analyses. In addition, the −NH_2_ group at 7N of SDZ could be oxidized to −NO_2_ via nitrification to form byproduct P13, which subsequently underwent a “Smiles” type recombination upon removal of −SO_2_ to form byproduct P14 due to the large Fukui function values at the 8S, 9O, and 10O positions.^[^ ^51^ ^]^ Finally, these intermediates were decomposed to form small organic acids, CO_2_, and H_2_O.

**Figure 5:**
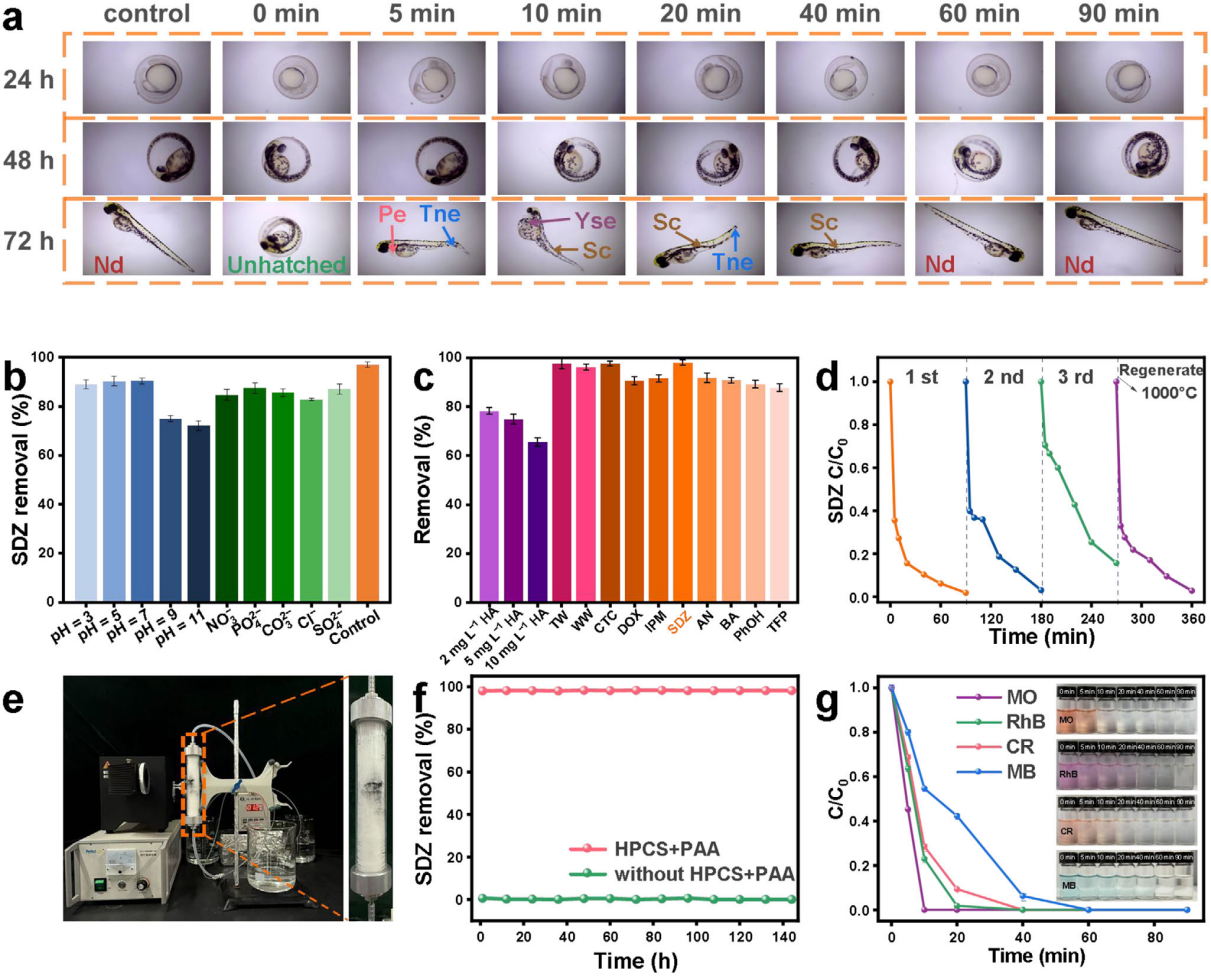
a) Development of zebrafish embryos with different reaction times in the HPCS + PAA system (Nd: Normal development; Pe: Pericardial edema; Tne: Tail non‐extension; Yse: Yolk sac edema; Sc: Spinal curvature). b) SDZ removal efficiency in the HPCS + PAA system under solution pH and different anions at 90 min. c) SDZ removal efficiency under different HA additions, across different water bodies (TW: tap water; WW: wastewater), and the removal efficiency for different antibiotics and aromatic ring compounds at 90 min in the HPCS + PAA system. d) SDZ degradation performances in the HPCS + PAA system under recycle of HPCS. e) Schematic of the continuous flow HPCS + PAA system unit. f) Performance of SDZ removal in a continuous flow HPCS + PAA system. Removal performance of HPCS + PAA system for g) different dyes. Experimental conditions: [HPCS] = 300 mg L^−1^, [PAA] = 227.3 µmol L^−1^, [antibiotics] = 1 mg L^−1^, [aromatic ring organics] = 1 mg L^−1^, [dyes] = 2 mg L^−1^, calcination temperature = 1000 °C, treatment time = 90 min, and pH 7.0. (Error bars: ± SD, *n* = 3).

The results of the developmental toxicity tests in zebrafish embryos are shown in **Figure** **5**a. During the first 72 h of embryonic development, zebrafish embryos exposed to water samples within 40 min of HPCS+PAA treatment exhibited pericardial edema (Pe), yolk sac edema (Yse), and development of a marked tail non‐extension (Tne) and spine curvature (Sc) compared with that in the control group. Zebrafish embryos developed normally and no developmental malformations were observed when exposed to water samples after 60 min of HPCS+PAA treatment. This suggested that SDZ was highly developmentally toxic to zebrafish embryos, and HPCS+PAA system was effective in reducing the toxicity of SDZ.

To evaluate the viability of the HPCS+PAA system, SDZ degradation performances were further assessed under different operational conditions. The degradation efficiency of SDZ remained ≈ 90% within 90 min treatment under both acidic and neutral conditions (Figures 5b; S22, Supporting Information), as well as in the presence of 0.1 mmol L^−1^ CO_3_^2−^, HPO_4_^2−^, SO_4_^2−^, NO_3_^−^, Cl^−^, Ca^2+^, and Mg^2+^ (Figures 5b; S23, Supporting Information). However, a slight decline in efficiency was observed under alkaline conditions (pH ≥ 9), which could be attributed to the base‐catalyzed decomposition of PAA into acetate and oxygen,^[^ ^1^, ^4^ ^]^ thereby reducing the availability of oxidant for activation. The confined thermal environment within the HPCS help to mitigate the influence of bulk pH variations, ensuring stable and robust performance across a practical pH range. The presence of natural organic matter, like humic acid (HA), had an inhibitory impact on SDZ degradation, and ≈75% of SDZ was still degraded within 90 min of treatment in the presence of 2 mg L^−1^ HA addition. In contrast, the addition of kaolinite (50 NTU) resulted in minimal disruption of the system, with SDZ removal efficiency remaining above 90%. The removal of SDZ from both tap water (TW) and secondary effluents from wastewater treatment plants (WW) exceeded 90% within 90 min of treatment, closely matching the efficiency observed in deionized water (Figures 5c and S24, Supporting Information). Although the SDZ degradation efficiency decreased to 85% within 90 min of treatment after three consecutive runs, more than 98% of SDZ was still degraded after the regeneration of HPCS under 1000 °C (Figure 5d). Furthermore, continuous‐flow experiments were performed to evaluate the long‐lasting robustness of the HPCS+PAA system, as shown in Figure 5e. More than 98% of the SDZ was removed every 12 h during 144 h of continuous operation, demonstrating the excellent stability of the HPCS+PAA system (Figure 5f). The temperature of industrial wastewater is closely related to the production process, and some industrial production processes discharge wastewater at high temperatures. Therefore, we evaluated the degradation performance of SDZ at various water temperatures. The kinetic constant of the SDZ degradation exhibited an upward trend with increasing water temperature. In detail, the SDZ degradation rate constant increased by 12 times as the water temperature rose from 40 to 80 °C in the HPCS+PAA system; however, it only increased by 3 times in the PCS+PAA system (Figure S25, Supporting Information). These results demonstrated that heat from wastewater could also induce heat‐confinement of HPCS, thus favoring contaminant degradation. In addition, the HPCS+PAA system exhibited great performances for removal of different antibiotics (chlortetracycline (CTC), doxorubicin (DOX), and imipenem (IPM)), different aromatic ring compounds (aniline (AN), benzoic acid (BA), phenol (PhOH), and tetrafluorophenol (TFP)), and different dyes (methyl orange (MO), congo red (CR), rhodamine B (RhB), and methylene blue (MB)), with efficiencies more than 90% within 90 min of treatment (Figures 5c,g; S26, Supporting Information). Finally, the energy efficiency of the HPCS photothermal system was quantitatively compared with that of conventional thermal PAA activation (Table S5, Supporting Information). Unlike bulk heating, which consumed energy to heat the entire solution, the HPCS confined energy to the catalytic sites via localized photothermal conversion. This approach resulted in over 4 times improvement in energy utilization efficiency compared to conventional heating, highlighting the exceptional energy economy of the “light‐gated thermal domains” by avoiding wasteful bulk heating. Thus, the heat‐confined HPCS catalyst exhibited outstanding stability and scalability, indicating its strong potential for practical applications.

## 3 Conclusion

In this study, a concept of “light‐gated thermal domains” was developed, in which confined heat hotspots within HPCS was successfully fabricated. The heat‐confined HPCS exhibited exceptional photo‐induced heating characteristics, outstanding redox capacity, remarkable optical response performance, and robust electron transfer capability. Thus, more than 98% of SDZ decomposed within 90 min in the HPCS+PAA catalytic system, which was significantly higher than that in the non‐confined system. The heat‐confined HPCS efficiently converted light energy into localized high temperatures, thereby enhancing the energy utilization efficiency, promoting electron density delocalization, and strengthening the interactions with the PAA molecules. These effects reduced the PAA activation energy barrier and enabled synergistic radical and nonradical oxidation pathways for pollutant degradation. The dual‐function adsorption‐activation sites on HPCS minimized ·OH diffusion distances, boosting catalytic activity. Zebrafish embryo toxicity assays showed that the HPCS+PAA system was effective in reducing SDZ toxicity. Furthermore, the scalability and practical viability of the HPCS+PAA system were validated via continuous‐flow experiments and the removal of various types of organic pollutants. This study revolutionizes the PAA activation approach via a “light‐gated thermal domains” strategy, underscoring its potential for water purification.

## 4 Experimental Section

Experimental details can be found in the Supporting Information.

## Conflict of Interest

The authors declare no conflict of interest.

## Supporting information



Supporting Information

## Data Availability

The data that support the findings of this study are available from the corresponding author upon reasonable request.
