# Supplementary material for: Light‐Gated Thermal Domains in Nano‐Lanterns: Confined Heat Hotspots Sparks Electron Localization for Water Purification
Source: Adv Sci (Weinh). 2025 Oct 30;13(3):e13730. doi: 10.1002/advs.202513730 (PMC12806286; doi:10.1002/advs.202513730)
Supplement: Supplementary file 1 — Supporting Information [file ADVS-13-e13730-s001.docx]

Supporting Information

**Light-Gated Thermal Domains in Nano-Lanterns: Confined Heat Hotspots Sparks Electron Localization for Water Purification**

*Miao Fang, Zhiyuan Ning, He Guo***,* *Xiaoteng Fan, Guodong Zhang, Qiuling Ma, Jian Zhou, Tiecheng Wang***, Sihui Zhan**

M. Fang, Z. Ning, G. Zhang, Q. Ma, J. Zhou, T. Wang

State Key Laboratory of Soil and Water Conservation and Desertification Control, College of Natural Resources and Environment, Northwest A&F University, Yangling, Shaanxi Province 712100, PR China

Email: wangtiecheng2008@126.com

H. Guo

College of Biology and the Environment

Nanjing Forestry University

Nanjing 210037, P.R. China

Email: [heguo@njfu.edu.cn](mailto:heguo@njfu.edu.cn)

X. Fan

College of Animal Science and Technology

Northwest A&F University

Yangling 712100, P.R. China

S. Zhan

School of Environmental Science & Engineering

Tianjin University

Tianjin 300350, P.R. China

Email: shzhan@tju.edu.cn

**26 Figures, 6 Tables**

**Experimental Section**

***Materials*:** Analytical grade ammonium fluoride (NH_4_F), tetraethyl silicate (TEOS), dopamine hydrochloride, peroxyacetic acid (PAA), sulfadiazine (SDZ), humic acid (HA), silver nitrate, tert-butanol (TBA), 2,4-hexadienoic acid (2,4-HD), triethanolamine (TEA), *p*-benzoquinone (*p*-BQ), anhydrous ethanol, chlortetracycline (CTC), doxorubicin (DOX), imipenem (IPM), aniline (AN), benzoic acid (BA), phenol (PhOH), tetrafluorophenol (TFP), methyl orange (MO), congo red (CR), rhodamine B (RhB), and methylene blue (MB) were bought from McLean Biochem. Technol. Co. Ltd. (Shanghai, China). Chromatographic grade methanol (MeOH), 5,5-dimethyl-1-pyrrolidinium-N-oxide (DMPO), dimethyl sulfoxide (DMSO), and 2,2,6,6-tetramethylpyridine (TEMP) were obtained from Sinopharm Chem. Reagent Co. Ltd (Nanjing, China).

***Synthesis of PCS and*** ***HPCS*:** PCS and HPCS were prepared using a hard-template method with high-temperature calcination. In detail, (1) ultrapure water (80 mL), anhydrous ethanol (19 mL), and NH_4_F solution (1 mL) were mixed and stirred for several minutes. (2) TEOS (1.5 mL) was added to the solution and stirred for 10 min. (3) dopamine hydrochloride (0.4 g) was added and stirred for 24 h. (4) The black precursor was centrifuged, washed, and freeze-dried for 48 h. (5) Afterward, the black precursor was calcined in a tube furnace under N_2_ atmosphere at different temperatures (i.e. 600°C, 700°C, 800°C, 900°C, 1000°C, and 1100°C) for 1 h at a rising rate of 5°C min^−1^. (6) The obtained catalysts were named as PCS-600°C, PCS-700°C, PCS-800°C, PCS-900°C, PCS-1000°C, and PCS-1100°C, respectively.

The prepared PCS catalysts were washed with 10% HF solution and neutralized with deionized water to remove the SiO_2_ template. The catalysts were freeze-dried for 24 h. The obtained samples were named as HPCS-600°C, HPCS-700°C, HPCS-800°C, HPCS-900°C, HPCS-1000°C, and HPCS-1100°C, respectively.

***Catalyst characterizations*:** The catalyst properties were examined by X-ray photoelectron spectroscopy (XPS, Thermo Scientific K-Alpha+, USA), Fourier transform infrared spectroscopy (FT-IR, Thermo Fisher Nicolet iS10, USA), Transmission electron microscopy (TEM, FEI Talos F200x, USA), Scanning electron microscopy (SEM, ZEISS Sigma 300, Germany), and Raman spectroscopy (Raman, Horiba LabRAM HR Evolution, Japan). The pore size distribution was determined using a pore size analyzer (Brunauer-Emmett-Teller (BET), Micromeritics ASAP 2460, USA). A UV-visible near spectrophotometer (UV/vis-IR, Perkinelmer Inc. Lambda 1050+, UK) was used to measure the absorbance of the materials.

The electrochemical properties of the materials were characterized using an electrochemical workstation (CHI 660E, China). Cyclic voltammetry (CV), linear scanning voltammetry (LSV), i-t curves, Mott-Schottky curves, and electrochemical impedance spectroscopy (EIS) were performed using an Electrochemical workstation (CHI 660E, China). Catalyst-coated FTO glass (20 mm × 20 mm × 1.6 mm; 14 Ω) functioned as the working electrode, the counter electrode was a platinum electrode, and the reference electrode was a saturated sugar electrode. First, 10 mg of the catalyst was dispersed in 300 µL of ultrapure water and 700 µL of anhydrous ethanol. After sonication for 10 min, 100 µL Nafion solution (5%, D520, DuPont) was added to the mixed suspension, and sonicated for 30 min. Then, a 10 µL drop of the mixed suspension was placed on the FTO electrode for natural drying. The electrolyte was a 0.05 M Na_2_SO_4_ solution. The EIS spectra were monitored in the frequency range of 0.001 Hz to 100 kHz. The start voltage of the LSV was set at 1 V and the termination voltage was set at −1 V. For Mott-Schottky, the start and termination voltages were set at −0.8 V and 0.8 V, respectively, with an amplitude of 0.01 V and a frequency of 1000. The i-t curve was initiated at 0.01 seconds and terminated at 160 seconds with a light chopping pattern of 0 s off, 10 s on, 30 s off, 40 s on, 60 s off, 70 s on, 90 s off, 100 s on, 120 s off, 130 s on, 150 s off, and 160 s on.

***Single-particle temperature simulation*:** Single-particle temperature simulations were carried out using ANSYS Fluent software to model single pore HPCS particles (*d* = 300 nm, wall thickness = 20 nm, average pore size = 4.7 nm, thermal conductivity = 500 W in a porous system (*d* = 70 μm, *h* = 70 μm)) under irradiation of temperature variations in the interior, shell, and exterior. Water transport is assumed to follow Poiseuille's law^[1]^. A 3D particle image model was obtained by the axisymmetric transformation of the 2D particles. The system was proposed to be scaled up by a factor of 500, owing to software limitations for nanoscale simulations. The outlet pressure was set at the top boundary. The side boundary was set as this drawback with a heat transfer coefficient of 5 W m^−2^ K^−1^. The lower boundary was set as an adiabatic boundary. The ambient temperature was 25℃. Plots were calculated before the start of the reaction (0 s) and 5400 s after the start of heat transfer.

***Catalytic performance evaluation*:** SDZ degradation experiments were carried out in a photochemical reactor. The reaction volume was 50 mL, and a 500 W Xenon lamp was applied as the reaction light source (CME-PC6). Initially, a certain mass of the catalyst was added to the SDZ solution and stirred at 1000 rpm. After adsorption equilibrium, 227.32 μmol L^−1^ PAA was added to the solution to initiate the reaction. At different times, 200 μL solution was sampled to determine the residual SDZ concentration.

The degradation kinetics were analyzed using a pseudo-first-order model. The observed rate constant (*K*_obs_, min^−1^) was determined by linear regression of ln(*C*_0_/*C*_t_) versus time (*t*) expressed as:

$\ln\text{(}\frac{\text{C}_{\text{0}}}{\text{C}_{\text{t}}}\text{) = }K_{\text{obs}}\text{ }\text{∙}\text{ }\text{t}$ (1)

where *C*_0_ (mg L^−1^) is the initial concentration of SDZ and *C*_t_ (mg L^−1^) is the concentration at the time *t* (min). Linear fitting was performed with a minimum correlation coefficient (*R*^2^) threshold of 0.95.

The SDZ content was monitored using High-performance liquid chromatography (HPLC, Agilent 1260). Reactive oxygen species (ROS) were detected using an electron spin resonance spectrometer (EPR, Bruker EMXplus-6/1, Germany) with TEMP or DMPO as trapping agents.

High-performance liquid chromatography-quadrupole time-of-flight high-resolution mass spectrometry (HPLC-MS, Thermo Scientific Ultimate 3000 UHPLC-Q Exactive) was used to identify the degradation intermediates. The concentration of SDZ was determined by an HPLC with a C_18_ column (150 × 4.6 mm, 5 μm). The detection wavelength was 265 nm. The mobile phase was a binary solution of 0.2% formic acid: acetonitrile (85%: 15%), and the flow rate was 1 mL min^−1^ with an injection volume of 10 µL. The temperatures of the column and oven were set at 35°C.

For HPLC-MS, the column temperature was 30°C and the injection volume was 10 μL. Thermo Scientific Q Exactive was used to identify the intermediates of SDZ in the ESI positive mode. The sheath gas rate was 40 arb, the auxiliary gas rate was 10 arb, the needle heating temperature was 350°C, the capillary temperature was 320°C, the spray voltage was 4.0 kV for positive ions, the S-lens was 50%, and the scan mode was Fullms/dd-ms2 top10. The scanning range was 70000 resolution and 50−600 m/z range at the first-level scanning and 17500 resolution at the second-level scanning.

***DFT calculations*:** The DFT, electrostatic potentials, and HOMO/LUMO orbitals of the SDZ molecules were analyzed using Gaussian software (version 16.0; b3lyp/6-31g (d)) and plotted using VMD software. During this period, Multiwfn software provided the means to establish Gaussian input files and obtain the Fukui function of the SDZ molecules^[2]^.

The Perdew-Burke-Ernzerhof exchange-correlation function with Grimme D3 dispersion correction was utilized for DFT calculations of the different catalysts^[3]^. The core-valence interactions were described using the projected augmented wave method^[4]^. The Brillouin zone integration for structural optimization was sampled using 1 × 1 × 1 Monkhorst-Pack grid k-points with an energy cutoff of 450 eV. The vacuum for both the lower and upper surfaces of the model was set at 10 A. Structural optimization was performed with energy and force convergence criteria setting at 1.0×10^−5^ eV and 0.02 eV A^−1^, respectively^[5]^.

***Zebrafish embryo toxicity assays:*** A zebrafish embryo bioassay was performed using wastewater samples collected at various oxidation treatment times (e.g., 0, 5, 10, 20, 40, 60, and 90 min). Wastewater samples were immediately transferred to sterile glass containers to prevent contamination. Adult male and female zebrafish (*Danio rerio*) reared under standard conditions (28.5 ± 0.5°C, 14 h light/10 h dark cycle) were used as broodstock. E3 medium was used for embryo cultivation. The E3 medium employed was prepared with 5 mmol L^−1^ NaCl, 0.17 mmol L^−1^ KCl, 0.33 mmol L^−1^ CaCl_2_, 0.33 mmol L^−1^ MgSO_4_, and 1 × methylene blue to inhibit fungal growth. Before use, the medium was autoclaved at 121°C for 20 min and cooled to room temperature. To obtain embryos, male and female pairs were separated in the breeding pool overnight and allowed to spawn naturally after the partitions were removed the following morning. Embryos were then collected and washed three times with culture water to remove debris and unfertilized eggs. Subsequently, 1 mL of the wastewater sample was taken and added to a 100 mL Petri dish, followed by the addition of 50 mL of zebrafish culture water as a nutrient solution. A blank control group was set up. The duration of embryo incubation was 72 h, and the morphological changes in the zebrafish embryos were continuously monitored. All procedures involving zebrafish were performed in strict accordance with the guidelines in the Zebrafish Book. The study protocol was approved by the Animal Ethics and Welfare Committee of Northwest A&F University (No. DK2022035) and all applicable provincial regulations (Shaanxi Province, No. 150, 2011).

***Catalyst recycling and regeneration procedure*:** Catalysts were regenerated after three reaction cycles using a comprehensive cleaning and thermal treatment process. The recovered catalysts were first filtered through a 0.45 μm aqueous polyethersulfone (PES) membrane, then thoroughly washed three times with ultrapure water and ethanol to remove both hydrophilic and hydrophobic residues. After washing, the materials were freeze-dried at −40°C for 48 h. After three recycling cycles, the dried catalysts were regenerated by anaerobic calcination in a tube furnace at 1000°C for 1 h under a nitrogen atmosphere. The mass recovery rate exceeded 90%.

***Continuous-flow experiments*:** During the continuous-flow experiments, a 500 W Xenon lamp was used to irradiate the filters (*d* = 20 mm, *L* = 120 mm) filled with 300 mg of HPCS. A constant-flow pump (HL-2B) supplied a 1 mg L^−1^ SDZ solution at a flow rate of 6 mL h^−1^ and a 226.32 μmol L^−1^ PAA solution at a flow rate of 6 mL h^−1^. The fluid was sampled every 12 h over a cumulative period of 144 h to measure the SDZ concentration.

***Degradation performance of different pollutants*:** Degradation experiments with different organic compounds were also conducted. For each experiment, 300 mg L^−1^ of catalyst was added to the solution containing different organic compounds (antibiotics: 1 mg L^−1^; aromatic ring compounds: 1 mg L^−1^; dyes: 2 mg L^−1^), and the mixture was stirred at 1000 rpm. After the adsorption equilibrium was achieved, 227.32 μmol L^−1^ of PAA was added to initiate the reaction. Samples were collected at different time intervals to determine the residual concentrations of organic pollutants.

***Calculation methods for energy consumption analysis*:** The total optical energy input into the HPCS+PAA system was calculated based on the light source parameters. The total incident optical energy (*E_light input_*, kWh) was given by:

$\text{E}_{\text{light input}} \text{= }\text{I }\text{∙}\text{ A }\text{∙}\text{ t }$ (2)

where *I* is the irradiance (light power density, kW m^−2^), *A* is the irradiated area (m^2^), and *t* is the total reaction time (h).

This optical energy input was similarly normalized by the solution volume (*V*, m^3^) to obtain the volumetric optical energy input (*E*_v, HPCS_, kWh m^−3^):

$\text{E}_{\text{v, HPCS}} \text{= }\text{E}_{\text{light input}}\text{ }\text{∕}\text{ }\text{V}\text{ }$ (3)

***Statistical analysis*:** All experimental data were derived from at least three independent replicates (*n* ≥ 3) unless otherwise specified. Data processing included the removal of technical outliers based on the Grubbs’ test (*α* = 0.05), though no significant outliers were identified in the presented datasets. Data were presented as mean ± standard deviation (SD) in all figures and tables.

The sample size (*n*) for each experiment was explicitly indicated in the corresponding figure captions. Significant differences between experimental groups were assessed using one-way analysis of variance (ANOVA) followed by Tukey’s post-hoc test for multiple comparisons, with a significance level set at *p* < 0.05. All statistical tests were two-sided. The assumptions of normality and homogeneity of variance were verified using the Shapiro-Wilk test and Levene’s test, respectively.

Statistical analyses were conducted using Excel 2021 (descriptive statistics) and Origin 2023 (linear regression for kinetics, graphing with mean ± SD and significance labels).

Figure S1. a) SEM and b) TEM images of PCS-1000°C. HAADF images and SAED patterns of c) HPCS-1000°C and d) PCS-1000°C. Elemental mappings of e) HPCS-1000°C and f) PCS-1000°C. EDS of g) HPCS-1000°C and h) PCS-1000°C

Figure S2. N_2_ adsorption-desorption isotherm and pore size distribution of HPCS-1000°C and PCS-1000°C

**Figure S3.** XPS survey of HPCS-1000°C and PCS-1000°C

**Figure S4.** The XPS spectra of O1s for HPCS catalysts prepared under a) 600°C, b) 700°C, c) 800°C, d) 900°C, e) 1000°C, and f) 1100°C.

**Figure S5.** Changes in solution temperature in the PAA oxidation system activated by HPCS prepared under different temperatures

**Figure S6.** Zeta potential of HPCS and PCS materials prepared under different temperatures. (*p* < 0.05, Error bars: ± SD, *n* = 3. The lowercase represents significant differences among different pyrolysis temperatures of the same catalyst).

**Figure S7.** SDZ degradation performances in PAA oxidation systems activated by PCS and HPCS catalysts prepared under a) 600°C, b) 700°C, c) 800°C, d) 900°C, e) 1000°C, and f) 1100°C. Experimental conditions: [CSs] = 200 mg L^−1^, [PAA] = 227.3 μmol L^−1^, [SDZ] = 10 mg L^−1^, treatment time = 60 min, and pH 7.0. (Error bars: ± SD, *n* = 3).

**Figure S8.** a) SDZ degradation performances and b) degradation kinetics in the PAA oxidation system activated by HPCS prepared at different temperatures. Experimental conditions: [CSs] = 200 mg L^−1^, [PAA] = 227.3 μmol L^−1^, [SDZ] = 10 mg L^−1^, treatment time = 60 min, and pH 7.0. (*p* < 0.05, Error bars: ± SD, *n* = 3).

**Figure S9.** a) Decomposition performances and b) decomposition kinetics of PAA by HPCS prepared at different temperatures. Experimental conditions: [CSs] = 200 mg L^−1^, [PAA] = 227.3 μmol L^−1^, [SDZ] = 1 mg L^−1^, treatment time = 60 min, and pH 7.0. (*p* < 0.05, Error bars: ± SD, *n* = 3).

**Figure S10.** a) SDZ degradation performances and b) degradation kinetics in the PAA oxidation system activated by PCS and HPCS under different SDZ initial concentrations. Experimental conditions: [CSs] = 200 mg L^−1^, [PAA] = 227.3 μmol L^−1^, calcination temperature = 1000°C, treatment time = 90 min, and pH 7.0. (*p* < 0.05, Error bars: ± SD, *n* = 3. The lowercase represents significant differences between different catalysts at the same SDZ dosage).

**Figure S11.** a) SDZ degradation performances and b) degradation kinetics in the PAA oxidation system activated by PCS and HPCS of different dosages. Experimental conditions: [PAA] = 227.3 μmol L^−1^, [SDZ] = 1 mg L^−1^, calcination temperature = 1000°C, treatment time = 90 min, and pH 7.0. (*p* < 0.05, Error bars: ± SD, *n* = 3. The lowercase represents significant differences between different catalysts at the same CNs dosage).

**Figure S12.** a) Decomposition performances and b) decomposition kinetics of PAA in different systems under irradiation. Experimental conditions: [CSs] = 300 mg L^−1^, [PAA] = 227.3 μmol L^−1^, calcination temperature = 1000°C, [SDZ] = 1 mg L^−1^, treatment time = 90 min, and pH 7.0. (*p* < 0.05, Error bars: ± SD, *n* = 3).

**Figure S13.** a) SDZ degradation performances and b) degradation kinetics in the PAA oxidation system activated by PCS and HPCS under different conditions. Experimental conditions: [CSs] = 300 mg L^−1^, [PAA] = 227.3 μmol L^−1^, calcination temperature = 1000°C, [SDZ] = 1 mg L^−1^, treatment time = 90 min, and pH 7.0. (*p* < 0.05, Error bars: ± SD, *n* = 3).

**Figure S14.** a) Decomposition performances and b) decomposition kinetics of PAA in different systems under different conditions. Experimental conditions: [CSs] = 300 mg L^−1^, [PAA] = 227.3 μmol L^−1^, calcination temperature = 1000°C, [SDZ] = 1 mg L^−1^, treatment time = 90 min, and pH 7.0. (*p* < 0.05, Error bars: ± SD, *n* = 3).

**Figure S15.** SDZ degradation performances in the HPCS+PAA system under the addition of different scavengers (a: TBA; b: MeOH; c: Mn^2+^; d: 2,4-HD; e: TBA; f: BQ; g: FFA; h: AgNO_3_; i: TEA. Experimental conditions: [HPCS] = 300 mg L^−1^, calcination temperature = 1000°C, [PAA] = 227.3 μmol L^−1^, [SDZ] = 1 mg L^−1^, treatment time = 90 min, and pH 7.0. (Error bars: ± SD, *n* = 3).

**Figure S16.** a) Degradation performances and b) degradation kinetics of SDZ in different systems under O_2_-rich and anaerobic conditions. Experimental conditions: [CSs] = 300 mg L^−1^, calcination temperature = 1000°C, [PAA] = 227.3 μmol L^−1^, [SDZ] = 1 mg L^−1^, treatment time = 90 min, and pH 7.0. (*p* < 0.05, Error bars: ± SD, *n* = 3).

**Figure S17.** a,b) SDZ degradation performances in the PCS+PAA system under the addition of different scavengers. c) SDZ degradation kinetics under different ROS scavengers. Experimental conditions: [PCS] = 300 mg L^−1^, calcination temperature = 1000°C, [PAA] = 227.3 μmol L^−1^, [SDZ] = 1 mg L^−1^, treatment time = 90 min, and pH 7.0. (*p* < 0.05, Error bars: ± SD, *n* = 3).

**Figure S18.** Front view and top view of a) HPCS model and b) PCS model.

**Figure S19.** a) Molecular structural formula of SDZ. b) Electrostatic potential energy distribution of SDZ. c) HOMO-LUMO orbit of SDZ.

**Figure S20.** Mass spectrometry molecule-mass plot of byproducts

**Figure S21.** Possible decomposition processes of SDZ in the HPCS+PAA oxidation system

**Figure S22.** SDZ degradation performances in the HPCS+PAA system under different solution pH. Experimental conditions: [HPCS] = 300 mg L^−1^, [PAA] = 227.3 μmol L^−1^, calcination temperature = 1000°C, treatment time = 90 min, and pH 7.0. (Error bars: ± SD, *n* = 3).

**Figure S23.** SDZ degradation performances in the HPCS+PAA system under different anions and cations. Experimental conditions: [HPCS] = 300 mg L^−1^, [PAA] = 227.3 μmol L^−1^, calcination temperature = 1000°C, treatment time = 90 min, and pH 7.0. (Error bars: ± SD, *n* = 3).

**Figure S24.** SDZ degradation performances in the HPCS+PAA system under different HA addition, 50 NTU Kln, and different water bodies (Kln: kaolinite; TW: tap water; WW: wastewater). Experimental conditions: [HPCS] = 300 mg L^−1^, [PAA] = 227.3 μmol L^−1^, calcination temperature = 1000°C, and pH 7.0. (Error bars: ± SD, *n* = 3).

**Figure S25.** SDZ degradation performances in the different systems under different water temperatures. Experimental conditions: [CSs] = 300 mg L^−1^, [PAA] = 227.3 μmol L^−1^, calcination temperature = 1000°C, treatment time = 90 min, and pH 7.0. (*p* < 0.05, Error bars: ± SD, *n* = 3).

**Figure S26.** HPCS+PAA system for different antibiotics and aromatic ring compounds degradation. Experimental conditions: [HPCS] = 300 mg L^−1^, [PAA] = 227.3 μmol L^−1^, [antibiotics] = 1 mg L^−1^, [aromatic ring organics] = 1 mg L^−1^, calcination temperature = 1000°C, and pH 7.0. (Error bars: ± SD, *n* = 3).

**Table S1.** Elemental analysis of PCS-1000°C and HPCS-1000°C

| Samples | C (%) | O (%) | N (%) | Si (%) | BET (m^2^ g^−1^) |
| --- | --- | --- | --- | --- | --- |
| PCS-1000°C | 17.51 | 45.57 | 0.57 | 36.35 | 125.0 |
| HPCS-1000°C | 73.21 | 24.62 | 0.73 | 1.44 | 683.3 |

**Table S2.** Relationship between kinetic constants of SDZ degradation and PAA activation with oxygen vacancies density

| Reaction system | O_v_ density  (%) | *K*_obs_ of SDZ degradation (min^−1^) | *K*_obs_ of PAA activation  (min^−1^) |
| --- | --- | --- | --- |
| HPCS-600°C+PAA | 2.7 | 0.0049 | 0.0029 |
| HPCS-700°C+PAA | 19.7 | 0.0118 | 0.0032 |
| HPCS-800°C+PAA | 20.3 | 0.0134 | 0.0052 |
| HPCS-900°C+PAA | 21.4 | 0.0153 | 0.0056 |
| HPCS-1000°C+PAA | 34.5 | 0.0240 | 0.0090 |
| HPCS-1100°C+PAA | 19.8 | 0.0120 | 0.0032 |
| Linear relationship | − | y=0.0595x+0.0019  (*R*^2^ = 0.76) | y=0.0595x+0.0019  (*R*^2^ = 0.76) |

**Table S3.** Comparison of catalytic performances of various catalysts on oxidant activation

| Catalysts | Oxidants | Pollutants | Degradation rate (%) | Reaction rate constant (min^−1^) | Effect of pH or anion | References |
| --- | --- | --- | --- | --- | --- | --- |
| **HPCS** | **PAA/Vis** | **sulfadiazine** | **95** | **0.1744** | **Slightly** | **This study** |
| TM | PAA | sulfadiazine | 75 | 0.0185 | Obviously | [6] |
| PSBCs-500 | PAA | acetaminophen | 70 | 0.0082 | Slightly | [7] |
| AC600 | PAA | sulfamethoxazole | 90 | 0.0191 | Moderately | [8] |
| PAC | PAA | sulfamethoxazole | 70 | 0.0053 | Moderately | [9] |
| Fe_3_O_4_@ZIFs | PAA | sulfamethoxazole | 65 | 0.1275 | Slightly | [10] |
| MoS_2_@TCN-S | PMS/Vis | tetracycline | 80 | 0.0040 | Slightly | [11] |
| Bi_2_MoO_6_ | PMS/Vis | sulfapyridine | 55 | 0.0176 | Obviously | [12] |
| CW/4Co | PMS/Vis | [tetracycline](https://www.sciencedirect.com/topics/chemistry/tetracycline) | 60 | 0.0314 | Obviously | [13] |
| TiO_2_ | PDS/Vis | benzophenone-3 | 60 | 0.0093 | Moderately | [14] |

**Table S4.** Analysis of quenching effects on SDZ degradation kinetics

| Quenching conditions | Targeted species | Apparent contribution (%) | Interpretation & pathway classification |
| --- | --- | --- | --- |
| Control (*K*_0_) | None | *–* | Baseline rate constant |
| TEA | *h^+^* | ~96.0 | Major non-radical pathway |
| FFA | ^1^O_2_ | ~89.7 | Significant non-radical pathway |
| MeOH | ·OH, R-O· | ~95.6 | Major radical pathway |
| TBA and others | R-O· (e.g., CH_3_O_2_·) | ~72.1 | Important radical pathway |
| BQ | ·O_2_^–^ | ~92.6 | Radical precursor |
| AgNO_3_ | *e^–^* | ~13.2 | Confirms *h^+^* dominance |

**Table S5.** Organic molecules screened by HPLC-MS

| Number | Mass | Chemical formula | Structural formula |
| --- | --- | --- | --- |
| SDZ | 251.058 | C_10_H_10_N_4_O_2_S |  |
| P1 | 96.056 | C_4_H_5_N_3_ |  |
| P2 | 174.184 | C_5_H_7_NO_3_S |  |
| P3 | 187.097 | C_10_H_10_N_4_ |   OR   |
| P4 | 227.174 | C_8_H_7_N_3_O_5_ |  |
| P5 | 242.153 | C_8_H_6_N_3_O_6_ |   OR   |
| P6 | 94.029 | C_6_H_7_N |  |
| P7 | 118.086 | C_4_H_6_O_4_ |  |
| P8 | 265.063 | C_10_H_10_N_4_O_3_S |   OR   |
| P9 | 279.054 | C_11_H_10_N_4_O_3_S |  |
| P10 | 215.059 | C_7_H_10_N_4_O_2_S |  |
| P11 | 111.023 | C_4_H_5_N_3_O |  |
| P12 | 60.081 | CH_5_N_3_ |  |
| P13 | 281.033 | C_10_H_8_N_4_O_4_S |  |
| P14 | 217.104 | C_10_H_8_N_4_O_2_ |  |

**Table S6.**Energy Input and Utilization in traditional thermal activation vs. HPCS photothermal systems

| Parameters | Traditional thermal activation system^[15]^ | HPCS+PAA photothermal system | Comparison notes |
| --- | --- | --- | --- |
| Energy input form | Electrical | Optical | _ |
| Heating mode | Bulk Heating | Localized Heating | The HPCS method was inherently energy-saving. |
| Input energy consume | 71 kWh m^−3^ | 187.5 kWh m^−3^ | Directly comparing input energy consumption was meaningless |
| Reaction rate constant | 1 × (benchmark) | 11 × | The HPCS system was highly efficient |
| Effective energy consumption (same effect) | High | Low | This was the key strength. |

**SI References**

1. T. S. Zaripov, O. Rybdylova, S. S. Sazhin, *Int. Commun. Heat Mass* **2018**, *97*, 85-91.
2. T. Lu, F. Chen, *J. Comput. Chem.* **2012**, *33*, 580-592.
3. J. P. Perdew, K. Burke, M. Ernzerhof, *Phys. Rev. Let.* **1996**, *77*, 3865-3868.
4. G. Kresse, D. Joubert, *Phys. Rev. B* **1999**, 59, 1758-1775.
5. P. E. Blöchl, *Phys. Rev. B* **1994**, *50*, 17953-17979.
6. Z. Chen, L. He, S. Shen, D. Zhang, Z. Chen, L. Wu, L. Yang, *Prog. Nat. Sci.* **2023**, *33*, 458-466.
7. F. Wu, X. Yue, Y. Xu, J. Gao, C. Zhang, Z. Li, H. Zhang, *Sep. Purif. Technol.* **2025**, *353*, 128150.
8. C. Dai, S. Li, Y. Duan, K. H. Leong, S. Liu, Y. Zhang, L. Zhou, Y. Tu, *Water Res.* **2022**, *216*, 118347.
9. Z. Wang, Z. Chen, Q. Li, J. Wang, L. Cao, Y. Cheng, S. Yu, Z. Liu, Y. Chen, S. Yue, J. Ma, P. Xie, *Environ. Sci. Technol.* **2023**, *57*, 10478-10488.
10. Y. Guo, C. Zhou, X. Lv, S. Du, M. Sui, *Sep. Purif. Technol.* **2025**, *354*, 128729.
11. B. An, J. Liu, B. Zhu, F. Liu, G. Jiang, X. Duan, Y. Wang, J. Sun, *Chem. Eng. J.* **2023**, *478*, 147344.
12. J. Yang, T. Xie, Y. Mei, J. Chen, H. Sun, S. Feng, Y. Zhang, Y. Zhao, J. Wang, X. Li, J. He, H. Chen, *Appl. Catal. B: Environ.* **2023**, *339*, 123149.
13. R. Chen, H. Zhang, Y. Dong, H. Shi, *J. Mater. Sci. Technol.* **2024**, *170*, 11-24.
14. C. Lu, R. Song, J. Wang, K. Liu, T. Fu, R. Tang, L. Jiang, Z. Tong, H. Zhang, *Sep. Purif. Technol.* **2023**, *310*, 123168.
15. Y. Li, Q. Zhang, Y. Chong, W. H. Huang, C. L. Chen, X. Jin, G. Chen, Z. Fan, Y. Qiu, D. Ye, Environmental Science & Technology, *Environ. Sci. Technol.* **2024**, *58*, 5153-5161.
